# Virulence gene profiles of *Arcobacter* species isolated from animals, foods of animal origin, and humans in Andhra Pradesh, India

**DOI:** 10.14202/vetworld.2017.716-720

**Published:** 2017-06-30

**Authors:** M. Soma Sekhar, S. R. Tumati, B. K. Chinnam, V. S. Kothapalli, N. Mohammad Sharif

**Affiliations:** 1 Department of Veterinary Public Health and Epidemiology, NTR College of Veterinary Science, Gannavaram, Andhra Pradesh, India; 2 Department of Veterinary Microbiology, NTR College of Veterinary Science, Gannavaram, Andhra Pradesh, India; 3 Department of Veterinary Microbiology, College of Veterinary Science, Tirupati, Andhra Pradesh, India

**Keywords:** *Arcobacter*, *Arcobacter butzleri*, *Arcobacter cryaerophilus*, *Arcobacter skirrowii*, polymerase chain reaction, virulence genes

## Abstract

**Aim::**

This study aimed to detect putative virulence genes in *Arcobacter* species of animal and human origin.

**Materials and Methods::**

A total of 41 *Arcobacter* isolates (16 *Arcobacter butzleri*, 13 *Arcobacter cryaerophilus*, and 12 *Arcobacter skirrowii*) isolated from diverse sources such as fecal swabs of livestock (21), raw foods of animal origin (13), and human stool samples (7) were subjected to a set of six uniplex polymerase chain reaction assays targeting *Arcobacter* putative virulence genes (*ciaB*, *pldA*, *tlyA*, *mviN*, *cadF*, and *cj1349*).

**Results::**

All the six virulence genes were detected among all the 16 *A. butzleri* isolates. Among the 13 *A. cryaerophilus* isolates, *cadF, ciaB*, *cj1349, mviN*, *pldA*, and *tlyA* genes were detected in 61.5, 84.6, 76.9, 76.9, 61.5, and 61.5% of isolates, respectively. Among the 12 *A. skirrowii* isolates, *cadF, ciaB*, *cj1349, mviN*, *pldA*, and *tlyA* genes were detected in 50.0, 91.6, 83.3, 66.6, 50, and 50% of isolates, respectively.

**Conclusion::**

Putative virulence genes were detected in majority of the *Arcobacter* isolates examined. The results signify the potential of *Arcobacter* species as an emerging foodborne pathogen.

## Introduction

The genus *Arcobacter* encompasses a group of Gram-negative, fastidious, nonspore forming, motile, spirally curved rods of the family *Campylobacteraceae* [1]. *Arcobacter* species differ from *Campylobacter* species in their aerotolerance and growth at 15°C [2]. In contrast with the acceptance of *Escherichia coli*, *Salmonella*, and *Campylobacter* species as the main foodborne pathogens, the reports dealing with the association of *Arcobacter* species are limited. Over the past few years, studies regarding the isolation of *Arcobacter* species from animals, raw foods of animal origin, and diarrheic humans signify the potential food safety concern associated with them [3-5]. Among several *Arcobacter* species, *Arcobacter butzleri*, *Arcobacter cryaerophilus*, and *Arcobacter skirrowii* are reported to have veterinary and public health significance [2]. Furthermore, the presence of virulence genes and its cytopathogenic effect on *in vitro* cell lines resulted in the categorization of *A. butzleri* as a “serious hazard” to human health by the International Commission on Microbiological Specifications for Foods [6].

Putative virulence determinants such as *ciaB* (encodes *Campylobacter jejuni* invasion antigen B that contributes to host cell invasion), *mviN* (encodes virulence factor, inner membrane protein required for peptidoglycan biosynthesis), *pldA* (encodes outer membrane phospholipase A associated with lysis of erythrocytes), *tlyA* (the hemolysin gene), and *cadF* and *cj1349* (encodes fibronectin-binding proteins which promote the binding of bacteria to intestinal cells) were reported to be present in *A. butzleri* (RM4018) genome [7]. In addition, *cadF* (*Campylobacter* adhesion to fibronectin) protein also induces the internalization of bacterial cells by the activation of GTPases [7]. Despite increasing reports of the association of *Arcobacter* species with livestock and human diseases, the mechanisms of pathogenicity of *Arcobacter* species are still poorly understood [8,9].

Perusal of the available literature revealed a lack of information on virulence gene profiles of *Arcobacter* species of animal and human origin in India. The present study was carried out to characterize virulence gene profiles of *Arcobacter* species isolated from animals, foods of animal origin, and human sources in Andhra Pradesh, India.

## Materials and Methods

### Ethical approval

This work does not require ethical approval as we have collected fecal swabs after defecation.

### Reference strain

The reference strain *A. butzleri* (ATCC 49616) used in the present study was obtained from the Division of Veterinary Public Health, Indian Veterinary Research Institute, Izatnagar.

### Bacterial isolates

A total of 41 *Arcobacter* isolates recovered from diverse sources such as fecal swabs of livestock (21), raw foods of animal origin (13), and human stool samples (7) were used in this study. The identification of each isolate was carried out using the following tests: Gram-staining (Gram-negative, short ‘S’ shaped rods), dark-field microscopy (corkscrew motility), oxidase (positive), catalase (positive), nitrate reduction (positive), and hippurate hydrolysis (negative) [10]. Further, all the 41 isolates were confirmed at genus level as *Arcobacter* by genus-specific polymerase chain reaction (PCR) targeting 16S rRNA gene [11] and at species level as *A. butzleri* (16), *A. cryaerophilus* (13), and *A. skirrowii* (12) by multiplex PCR targeting 16S and 23S rDNA [12]. *Arcobacter* isolates from fecal swabs of livestock include those from pigs (8), chicken (6), turkey (2), cattle (2), sheep (2), and duck (1). *Arcobacter* isolates from raw foods of animal origin include those from chicken (5), pork (4), milk (2), and mutton (2). *Arcobacter* isolates from human stool samples include those from farm workers of pig/poultry (3), veterinary students (2), and diarrheic humans (2). Whole-cell DNA was extracted by boiling and snap chilling method [4]. The absorbance of the DNA at wavelengths 260 and 280 nm was measured using Nanodrop (Thermo Scientific, USA).

### Detection of putative virulence genes by PCR

All the 41 *Arcobacter* isolates were subjected to a set of six uniplex PCR assays for the detection of six putative virulence genes (*ciaB*, *pldA*, *tlyA*, *mviN*, *cadF*, and *cj1349*) [13]. Primer sequences, expected amplicon sizes, and virulence gene details are listed in Table-1. A preliminary gradient PCR was conducted with DNA of *A. butzleri* (ATCC 49616) which carried all the six putative virulence genes to assess the correct annealing temperature. With annealing temperatures lower than 56.0°C (for *ciaB, cj1349*, and *mviN* genes) and 58.0°C (for *cadF, pldA*, and *tlyA* genes), lower molecular weight products were detected in addition to expected amplicons. Thus, annealing temperatures of 56.0°C (for amplification of *ciaB, cj1349*, and *mviN* genes) and 58.0°C (for *cadF, pldA*, and *tlyA* genes) were optimized for future PCR reactions. All the six PCR assays were carried out in Eppendorf thermal cycler (USA) with a heated lid.

**Table-1:** Oligonucleotide primers used for detection of *Arcobacter* putative virulence genes.

Primer/Target gene	Virulence factor	Nucleotide sequence (5’ 3’)	Amplicon size (bp)
*cadF*	Fibronectin-binding proteins	TTACTCCTACACCGTAGT	283
		AAACTATGCTAACGCTGGTT	
*ciaB*	Invasion antigen B	TGGGCAGATGTGGATAGAGCTTGGA	284
		TAGTGCTGGTCGTCCCACATAAAG	
*cj1349*	Fibronectin-binding proteins	CCAGAAATCACTGGCTTTTGAG	659
		GGGCATAAGTTAGATGAGGTTCC	
*mviN*	Virulence factor	TGCACTTGTTGCAAAACGGTG	294
		TGCTGATGGAGCTTTTACGCAAGC	
*pldA*	Phospholipase A	TTGACGAGACAATAAGTGCAGC	293
		CGTCTTTATCTTTGCTTTCAGGGA	
*tlyA*	Hemolysin	CAAAGTCGAAACAAAGCGACTG	230
		TCCACCAGTGCTACTTCCTATA	

The PCR assays for *ciaB, cj1349*, and *mviN* genes were optimized in 25 µl reaction mixture containing 2 µl of DNA template; *Taq* buffer (10×) - 2.50 μl; dNTP mix (10 mM) - 1 μl; MgCl_2_ (25 mM) - 0.75 μl; forward primer (10 pmol/μl) - 1.50 μl; reverse primer (10 pmol/μl) - 1.50 μl; *Taq* DNA polymerase (1 U/μl) - 1 μl; and nuclease free water - 14.75 μl, under the following standardized cycling conditions: initial denaturation at 94°C for 5 min, 30 cycles of denaturation at 94°C for 1 min, annealing at 56°C for 45 s, elongation at 72°C for 1 min, and final elongation at 72°C for 10 min and hold at 4°C.

The PCR assays for *cadF, pldA*, and *tlyA* genes were optimized in 25 µl reaction mixture containing 2 µl of DNA template; *Taq* buffer (10×) - 2.50 μl; dNTP mix (10 mM) - 1 μl; MgCl_2_ (25 mM) - 1.5 μl; forward primer (10 pmol/μl) - 1.0 μl; reverse primer (10 pmol/μl) - 1.0 μl; *Taq* DNA polymerase (1 U/μl) - 1 μl; and nuclease free water - 15.0 μl, under the following standardized thermal cycling conditions: initial denaturation at 94°C for 5 min, 30 cycles of denaturation at 94°C for 1 min, annealing at 58°C for 45 s, elongation at 72°C for 1 min, and final elongation at 72°C for 10 min and hold at 4°C.

## Results and Discussion

**Figure-1:**
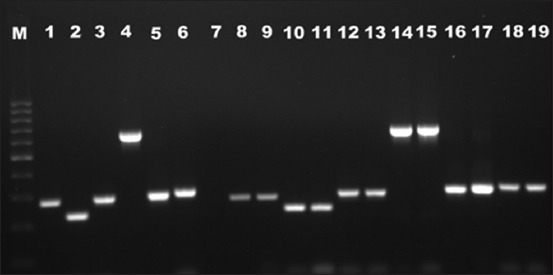
Gel photograph of polymerase chain reaction assays targeting *Arcobacter* putative virulence genes; Lane M: Molecular weight marker (100 bp); L1-6: Positive control of *Arcobacter butzleri* (ATCC 49616) carrying all 6 putative virulence genes, i.e., *cadF* (283 bp), *tlyA* (230 bp), *ciaB* (284 bp), *cj1349 (*659 bp), *pldA* (293 bp), and *mviN* (294 bp); L7: Negative control; L8: *A. butzleri* isolate with *cadF* (283 bp) gene; L9: *Arcobacter cryaerophilus* isolate with *cadF* (283 bp) gene; L10: *A. butzleri* isolate with *tlyA* (230 bp) gene; L11: *Arcobacter skirrowii* isolate with *tlyA* (230 bp) gene; L12: *A. butzleri* isolate with *ciaB* (284 bp) gene; L13: *A. cryaerophilus* isolate with *ciaB* (284 bp) gene; L14: *A. butzleri* isolate with *cj1349 (*659 bp) gene; L15: *A. skirrowii* isolate with *cj1349 (*659 bp) gene; L16: *A. butzleri* isolate with *pldA* (293 bp) gene; L17: *A. cryaerophilus* isolate with *pldA* (293 bp) gene; L18: *A. butzleri* isolate with *mviN* (294 bp) gene; L19: *A. skirrowii* isolate with *mviN* (294 bp) gene.

Putative virulence genes were detected in majority of screened *Arcobacter* isolates. The *ciaB* gene was predominant gene detected (92.6%, 38/41 isolates), followed by *cj1349 (*87.8%, 36/41), *mviN* (82.9%, 34/41), *cadF* (73.1%, 30/41), *pldA* (73.1%, 30/41), and *tlyA* (73.1%, 30/41) (Figure-1 and Table-2). All the 16 *A. butzleri* isolates carried all six putative virulence genes. None of the 13 *A. cryaerophilus* and 12 *A. skirrowii* isolates possessed all the six virulence genes. The present results were in accordance with the previous studies from Berlin [9] and Belgium [13] where 100% prevalence of these putative virulence genes in *A. butzleri* strains was reported.

**Table-2:** Putative virulence-associated genes detected in *Arcobacter* isolates using PCR.

Species and source	Number of strains examined	Number of strains generating specific gene amplicon					

		*cadF*	*ciaB*	*cj1349*	*mviN*	*pldA*	*tlyA*
*A. butzleri*							
Poultry feces	2	2	2	2	2	2	2
Pig feces	2	2	2	2	2	2	2
Cattle feces	1	1	1	1	1	1	1
Chicken meat	2	2	2	2	2	2	2
Pork	1	1	1	1	1	1	1
Milk	1	1	1	1	1	1	1
Veterinary students	2	2	2	2	2	2	2
Farm workers	3	3	3	3	3	3	3
Diarrheic humans	2	2	2	2	2	2	2
Total (%)	16	16 (100)	16 (100)	16 (100)	16 (100)	16 (100)	16 (100)
*A. cryaerophilus*							
Poultry feces	2	1	2	2	2	1	1
Pig feces	3	1	2	2	2	2	2
Cattle feces	1	1	1	1	1	1	0
Chicken meat	3	2	3	3	2	2	2
Pork	3	2	2	1	2	2	2
Milk	1	1	1	1	1	0	1
Total (%)	13	8 (61.5)	11 (84.6)	10 (76.9)	10 (76.9)	8 (61.5)	8 (61.5)
*A. skirrowii*							
Poultry feces	5	3	5	4	4	2	3
Pig feces	3	1	3	2	1	2	1
Sheep feces	2	1	2	2	1	1	1
Mutton	2	1	1	2	2	1	1
Total (%)	12	6 (50)	11 (91.6)	10 (83.3)	8 (66.6)	6 (50)	6 (50)
Grand total (%)	41	30 (73.1)	38 (92.6)	36 (87.8)	34 (82.9)	30 (73.1)	30 (73.1)

PCR=Polymerase chain reaction, *A. butzleri=Arcobacter butzleri, A. cryaerophilus=Arcobacter cryaerophilus, A. skirrowii=Arcobacter skirrowii*

Among the 13 *A. cryaerophilus* isolates, *ciaB* (84.6%, 11/13 isolates), *cj1349 (*76.9%, 10/13), and *mviN* (76.9%, 10/13) genes were detected frequently, followed by *cadF* (61.5%, 8/13), *pldA* (61.5%, 8/13), and *tlyA* (61.5%, 8/13) genes. In a study from Chile, putative virulence genes such as *ciaB* (100%), *mviN* (100%), *tlyA* (66.7%), *pldA* (33.3%), and *cj1349 (*14.3%) were detected in *Arcobacter* species, and *cadF* gene was reported to be not detected in *Arcobacter* species [14]. Among 12 *A. skirrowii* isolates, *ciaB* (91.6%, 11/12 isolates), *cj1349 (*83.3%, 10/12), and *mviN* (66.6%, 8/12) genes were frequently detected, followed by *cadF* (50%, 6/12), *pldA* (50%, 6/12), and *tlyA* (50%, 6/12) genes. In a study from Chile, putative virulence genes such as *ciaB* (93.8%), *mviN* (81.3%), *tlyA* (68.8%), *cj1349 (*31.3%), *pldA* (25%), and *cadF* (12.5%) genes were reported to be detected in *Arcobacter* species [14]. Predominance of putative virulence genes in *A. butzleri* was noticed compared to *A. cryaerophilus* and *A. skirrowii*, as evidenced by detection of all the six putative virulence genes in all the 16 *A. butzleri* isolates, whereas none of the 13 *A. cryaerophilus* and 12 *A. skirrowii* isolates carried all the six virulence genes, which was in agreement with the previous studies [14,15]. This may indicate possibility of differential pathogenic behavior of *Arcobacter* species or higher genomic heterogeneity [13] or bias from the use of *A. butzleri* ATCC 49616 genome sequence only in designing the virulence gene primers [8].

In a study from Belgium [13], three *Arcobacter* type strains (i.e., *A. butzleri* LMG 10828, *A. cryaerophilus* LMG 10210, and *A. skirrowii* LMG 6621) were reported to carry nine putative *Campylobacter* virulence genes (*cadF*, *ciaB*, *cj1349*, *hecA*, *hecB*, *irgA*, *mviN*, *pldA*, and *tlyA*). In addition, no significant difference was noticed in *cadF* gene detected among different strains of *Arcobacter* isolated from humans, cattle, horses, sheep, dogs, and chicken [13]. However, the role of these putative virulence determinants or *Campylobacter* homologs in the pathogenicity *Arcobacter* species was still contradictory [9,16]. *Arcobacter* species were reported to have ability to adhere and invade the intestinal epithelial cells of host and induce inflammatory responses [17]. *Arcobacter* infection was reported to be associated with leak flux type of watery diarrhea resulting from epithelial barrier dysfunction [18], and adhesion, invasion and toxin production could be the mechanisms of *Arcobacter* pathogenicity [9,19].

## Conclusion

Food safety needs a thorough investigation of virulence properties of potentially emerging pathogenic bacteria in animals and foods of animal origin. The presence of *Arcobacter* strains carrying virulence markers in animals, humans and foods of animal origin have not yet been a subject of investigation in India. The present study reporting the presence of six virulence-associated genes of three emerging *Arcobacter* species isolated from animal and human sources adds to the significance of *Arcobacter* as an emerging foodborne pathogen with zoonotic potential. Further studies should focus on examining the interactions of *Arcobacter* species virulence factors with eukaryotic cells.

## Authors’ Contributions

MSS is the student worked for M.V.Sc thesis. SRT as a major guide and BKC and VSK as minor guides designed and supervised the research work. NMS collected the samples and executed the isolation. MSS was involved in the molecular characterization. The manuscript was drafted and revised by MSS and NMS under the guidance of SRT, BKC, and VSK. All authors read and approved the final manuscript.
